# ONT sequencing identifies a high prevalence of *crt* sensitive, triple mutant *dhfr* and single mutant *dhps* parasites within an ANC population in Nigeria

**DOI:** 10.3389/fgene.2024.1470156

**Published:** 2024-10-15

**Authors:** Adebanjo Jonathan Adegbola, Leonard Ndwiga, Kevin Wamae, Victor Osoti, Oluseye Oladotun Bolaji, Philip Bejon, Lynette Isabella Ochola-Oyier

**Affiliations:** ^1^ Faculty of Pharmacy, Obafemi Awolowo University, Ile-Ife, Nigeria; ^2^ Kenya Medical Research Institute (KEMRI)-Wellcome Trust Research Programme (KWTRP), Kilifi, Kenya

**Keywords:** malaria, chemoprevension, surveillance, genotyping, sequencing, resistance

## Abstract

**Background:**

Malaria in pregnancy is a major public health issue, particularly among vulnerable populations in malaria-endemic sub-Saharan African countries. To mitigate its risks, WHO recommends sulphadoxine-pyrimethamine (SP) for chemoprevention and artemisinin-based combination therapy (ACT) to treat uncomplicated *Plasmodium falciparum* malaria. These interventions have helped to alleviate the risk associated with malaria in pregnancy; however, in the context of the emergence of SP- and ACT-resistant *P. falciparum*, maintained efficacy is under threat. Molecular surveillance is a reliable tool to monitor the emergence of resistance where molecular markers are known. Thus, the objective of the study was to use a multiplexed amplicon Oxford Nanopore sequencing approach to assess the molecular markers for antimalarial resistance among pregnant women in Nigeria.

**Methods:**

Dried blood spots (DBS) were collected from pregnant women who received IPTp-SP at the enrollment and follow-up visits. *P. falciparum* genomic DNA was extracted by the Chelex^®^ method and *Pf*18S qPCR was used to detect parasite DNA in each sample. With nested PCR assays, fragments of *Pfdhps*, *Pfdhfr*, *Pfmdr1*, *Pfcrt*, *Pfk13* and *Pfama1* genes were amplified and multiplexed amplicon-based sequencing was conducted on the minION Oxford Nanopore Technology.

**Result:**

In total, 251 pregnant women were enrolled in the study and 457 DBS samples were collected. *P. falciparum* genomic DNA was detected in 12% (56/457) of the samples, 31 at baseline and the remaining during the follow-up visits. *Pfama1*, *pfk13*, *Pfdhps*, *Pfdhfr*, *Pfmdr1 and Pfcrt* were successfully sequenced in a single run. Notably, k13 artemisinin resistance mutations were absent, the frequencies of *Pfdhfr* and *Pfdhps* SP resistance haplotypes, **IRN** for pyrimethamine resistance and I**SG**KA/IA**G**KA associated with sulphadoxine resistance were 82% (36/44) and 64% (27/42), respectively, and the *Pfcrt* CV**IET** resistant haplotype was at approximately 22% (7/32).

**Conclusion and recommendations:**

Here a multiplexed amplicon-based ONT assay established that triple mutant *Pfdfhr*-IRN, double mutant *Pfdhps*-SG haplotypes and the chloroquine sensitive strain were prevalent among pregnant women in Nigeria.

## Background

Malaria remains a major public health issue, particularly in sub-Saharan Africa (SSA) where more than 90% of malaria cases were reported in 2022 (WHO, 2023a). Globally, out of the 249 million malaria cases reported in 2022, more than 90% were reported in sub-Saharan Africa (WHO, 2023c). Exposure to malaria poses a considerable level of risk among vulnerable populations, children under the age of five and pregnant women. In 2022, a global estimate of 12.7 million (36%) from 35.4 million pregnancies, were exposed to malaria infection during pregnancy. Nigeria contributes the highest burden to global malaria morbidity and mortality (WHO, 2023c; WHO, 2023b). The consequences of malaria infection in these groups include low birth weight, preterm birth, placental malaria, maternal anaemia and anaemia in infants (Tiono et al., 2009; Bakken and Iversen, 2021; Chua et al., 2021a; Chua et al., 2021b).

Thus, interventions are now recommended to mitigate or prevent the risks associated with malaria in pregnancy (MIP). WHO has sustained the use of sulphadoxine-pyrimethamine (SP) for intermittent preventive treatment (IPT) for all pregnant women (IPTp-SP) starting in the second trimester and artemisinin-based combination therapy (ACT) for treatment of uncomplicated falciparum malaria (Madanitsa et al., 2023; WHO, 2023a).

However, the efficacy of IPTp-SP and ACT is being threatened by increasing reports of the emergence of SP- and artemisinin-resistant malaria parasites. Current evidence shows that IPTp with either SP or dihydroartemisinin-piperaquine (DP) is associated with reduced maternal parasitaemia (Gutman et al., 2021). The use of IPTp-SP is non-inferior to DP (Kajubi et al., 2019). Although, in areas with high SP resistance, DP seemed to be superior to SP in reducing clinical malaria during pregnancy, this did not translate into better pregnancy outcomes (Desai et al., 2015; Roh et al., 2020; Gutman et al., 2021). Therefore, based on the WHO recommendation (WHO, 2023a), pregnant women across malaria-endemic SSA countries are to receive ≥3 IPTp-SP doses starting from the second trimester until delivery. However, despite the evidence of these benefits, the growing concern of the emergence of several mutations in the genes conferring resistance to SP and other antimalarial drugs across SSA is worrisome (Adegbola et al., 2023; Nana et al., 2023).

Mutations in the *Plasmodium falciparum* chloroquine resistance transporter (Pfcrt) and multidrug resistance 1 (*mdr1*) genes have been associated with chloroquine and amodiaquine resistance, respectively, particularly mutations at K76T and N86Y of *Pfcrt* and *Pfmdr1*, respectively (Fidock et al., 2000; Djimdé et al., 2001; Happi et al., 2006; Holmgren et al., 2007). SP-resistance is predominantly driven by a cumulative build-up of mutations in the *P. falciparum* dihydrofolate reductase (*Pfdhfr*) and dihydropteroate synthetase (*Pfdhps*) genes, which encode enzymes targeted by pyrimethamine and sulphadoxine, respectively. Polymorphisms causing amino acid substitutions at codons 437, 540 and recently 581 of *Pfdhps* were associated with sulphadoxine resistance, while mutations at codons 51, 59, 108 and 164 confer resistance to pyrimethamine (Plowe et al., 1996; Roper et al., 2004; Alifrangis et al., 2014). There are other emerging mutations in the *Pfdhps* gene 431V, 436A, 437G, 540E and 613S suggesting an escalation of increasingly resistant strains in Africa (Oguike et al., 2016; Osoti et al., 2022; Adegbola et al., 2023; Tarama et al., 2023). Recent reports have also shown mutations in the Kelch 13 gene and their association with slow parasite clearance following ACT treatment across African countries (Uwimana et al., 2020; Balikagala et al., 2021; Straimer et al., 2022).

To supplement the data on therapeutic efficacy study (TES), WHO also recommends further molecular analysis, parasite DNA sequencing or genotyping, for continuous monitoring of antimalarial resistance markers to map the occurrence and spread of antimalarial resistance in malaria-endemic settings. In addition, molecular analysis of *P. falciparum* genetic diversity is also considered a metric for transmission intensity. Establishing surveillance for antimalarial resistance and genetic diversity markers within a population requires cost-effective, high-throughput and scalable sequencing assays.

Illumina sequencing assays have been described for drug resistance and genetic diversity marker surveillance (Nag et al., 2017; Diakité et al., 2019; Wamae et al., 2022). However, the capacity and access to these sequencing assays remain limited in malaria-endemic SSA countries. Oxford Nanopore Technology (ONT) sequencing is a more portable and cost-effective sequencing platform that has been employed for genomic surveillance of pathogens such as SARS-CoV-2 (Tshiabuila et al., 2022), Ebola virus (Hoenen et al., 2016) and *Mycobacterium tuberculosis (Dippenaar et al., 2022)*. The aim of this study was to monitor the *P. falciparum* drug resistance markers and apical membrane antigen (*ama) 1 P. falciparum* genetic diversity using the ONT sequencing platform among pregnant women who were being monitored for IPTp-SP chemoprevention efficacy.

## Methods

### Study population

The study was conducted at the antenatal clinic, State Specialist Hospital, Ilesa from August 2022 to May 2023 as a prospective cohort study among pregnant women who were ≥18 years. Those who consented to participate in the study were enrolled at mid second trimester, around 18 weeks of gestation, blood samples were collected as dried blood spots (DBS) and a dose of IPTp-SP was provided at the enrollment. The first and second follow-up visits were scheduled on day 28 and 56 respectively, with blood collected as a DBS. IPTp-SP doses were provided to each enrollee at each antenatal visit as part of antenatal care without routine malaria screening in line with the national malaria guideline. The study protocol was granted ethics approval by the Health Research and Ethics Committees of Obafemi Awolowo University Teaching Hospital Complex (HREC-OAUTHC). Study participants consented to the collection of the DBS and biobanking of the samples.

### DNA extraction

Venous blood was collected and aliquoted on Whatman^®^ 903 Protein saver cards (Sigma-Aldrich, Inc. MO, United States) for the assessment of *P. falciparum* molecular markers of antimalarial resistance. DNA was extracted from all DBS collected at the baseline and follow-up visits using the Chelex^®^ method. From each sample, three punches, about 3.0 mm per punch, were cut into a sterile 1.5 mL Eppendorf^®^ tube. The 3 DBS discs were soaked overnight using 1 mL of 0.5% w/v saponin prepared in 1X phosphate-buffered saline (PBS) to lyse the red blood cells. Following saponin aspiration, the discs were washed in 1 mL 1X PBS, incubated at 4°C for 30 min, before aspirating the PBS. The washing step was done twice. To each tube, 100 µL of a solution of 6% w/v Chelex in DNase/RNase-free water was added and incubated for 30 min at 96°C. Subsequently, the tubes were centrifuged at 4,000 × g for 5 min 80 µL of the DNA-containing solution was transferred from each tube into 96 well plates and stored at −20°C for further analyses.

### Antimalarial drug resistance marker genotyping

A qPCR assay based on Pf18S (small unit) rRNA primer-probe amplification on the Quantstudio™ real-time PCR system was adopted for the detection of *P. falciparum* following reaction mix and PCR program previously described (Osoti et al., 2022). Thereafter, positive samples with a Ct ≤40 were genotyped using primer sets previously reported by Osoti et al. (2022) and nested PCR assays were carried out to amplify regions in the genes associated with antimalarial drug resistance [ *Pfdhfr/Pfdhps* (sulphadoxine-pyrimethamine), *PfK13* (artemisinin), *Pfmdr1* (lumefantrine, amodiaquine and mefloquine)] and *Pfama1* for genetic diversity. Pfama1 amplicons were also generated for the Pf3D7, Pf7G8 and PfHB3 laboratory controls. Similarly, a one-step PCR approach was used for *Pfcrt* (194 bp) associated with chloroquine resistance using high-fidelity Expand DNA polymerase. The thermal cycling conditions are as follows: 94°C for 2 min, 35 cycles of 94°C for 15 s, 52°C for 30 s, 72°C for 2 min for 35 cycles for extension and a final extension of 7 min at 72°C. Post-PCR, amplicons for each gene were confirmed on 1% (w/v) agarose gel electrophoresis stained with 5 µL of RedSafe nucleic acid staining solution. Successful PCR products from each sample were pooled into a well in preparation for ONT ligation and barcoding.

### Amplicon pooling and ONT multiplexing sequencing

PCR products from the five genetic markers with a positive agarose gel result were sorted and mixed to produce amplicon pools for each sample and in addition, for each of the laboratory controls Pf3D7, Pf7G8 and PfHB3. These pooled amplicons were purified using the 1.5X AMPure XP Beads (Beckman Coulter) to capture the different amplicon sizes ranging 194 bp–886 bp. The amplicon pools were then eluted in 30 µL of resuspension buffer and quantified using the Qubit™ double-strand DNA (dsDNA) high-sensitivity (HS) assay kit (Invitrogen, Q32851). Thereafter, 130 ng of each amplicon-pool was used to prepare ONT sequencing libraries using the Ligation Sequencing Kit (SQK-LSK114) based on the manufacturer’s protocol. The library was eluted in 15 µL Elution buffer and 50 ng of library was loaded on the SpotON R10.4.1 flow cell (FLO-MIN114). Prior to loading, 800 µL of priming mix was loaded into the flow cell through the priming port. Priming was completed by loading 200 µL of priming mix into the flow cell priming port. 75 µL of the prepared library was loaded into the flow cell via the SpotON sample port in a dropwise fashion and the sequencing was run for 48 h.

### Sequence data analysis

Sequence data extraction, quality control, and microhaplotype clustering were performed using SeekDeep v3.0.1 (Hathaway et al., 2018). Henceforth, we use the term “microhaplotype” to refer to the set of alleles found on a single DNA amplicon. We implemented SeekDeep’s default threshold of 250 reads as the minimum required read depth for each sample. A conservative minor allele frequency threshold of 5% was implemented to filter out less prevalent alleles unless such alleles were independently observed in other samples with a frequency exceeding 5%, enhancing the reliability of allele detection across the dataset. Chimeric reads were considered PCR artefacts and discarded. Allele frequencies per codon were calculated by dividing the number of reads for each allele by the total number of reads spanning that codon within each sample and expressed as percentages. This enabled us to quantify the genetic variability within each sample and allowed us to trace each individual. Infections were later classified into wildtype, mutant, or mixed. Wildtype infections contained alleles that matched the reference sequence; mutants contained mono-allelic variants that differed from the wildtype sequence, while mixed infections contained multiple variants at the same codon position. The relative frequency for each microhaplotype was calculated by dividing its count in the population by the total count of microhaplotypes and expressing it as a percentage. This approach helped us identify common and rare microhaplotypes, which can be crucial for understanding genetic diversity and evolution patterns. The complexity of infection (COI) was defined as the number of distinct microhaplotypes (varying at the nucleotide level) in each sample.

## Results

### Demographic features of the study population

In total, 251 pregnant women were screened. Among the pregnant women, 95 (38%), 82 (33%) and 74 (30%) were primigravida, secundigravida and multigravida, respectively (Table 1). Each received at least three IPTp-SP doses during their ANC visits. The average age and weight of the study participants were 27.8 ± 5.4 years and 66.2 ± 4.5 kg, respectively. The mean gestational age in weeks and Packed Cell Volume (%) were 21.2 ± 2.7 and 31.3 ± 3.5, respectively at enrolment. During the first contact, 212 pregnant women volunteered blood samples as DBS on 903 Whatman^®^ protein saver paper. Furthermore, the participants were followed up on days 28 and 56. However, during the follow-up visits, only 153 and 92 study participants returned to the clinic, respectively to provide the DBS samples for molecular analyses. This made a total collection of 457 DBS samples which had not been previously screened with microscopy or RDT.

**TABLE 1:** Demographic data of pregnant women recruited into the study.

Demographic features	All participants (n = 251)	Primigravida (n = 95)	Secundigravida (n = 82)	Multigravida (n = 74)
Age (years)	27.84 ± 5.43	24.6 ± 4.54	28.45 ± 4.73	31.46 ± 4.74
Weight (kg)	66.23 ± 11.04	65.14 ± 10.38	64.41 ± 10.9	69.71 ± 11.39
Gestational at enrollment (weeks)	21.19 ± 2.68	21.2 ± 2.42	20.91 ± 2.66	21.49 ± 3.01
PCV at enrollment (%)	31.3 ± 3.5	31.09 ± 3.35	30.92 ± 3.27	32.01 ± 3.88
Number of DBS samples collected				
At enrollment	212	76	74	62
At the first follow-up	153	56	45	52
At the second follow-up	92	40	28	24

### PCR results

**FIGURE 1:**
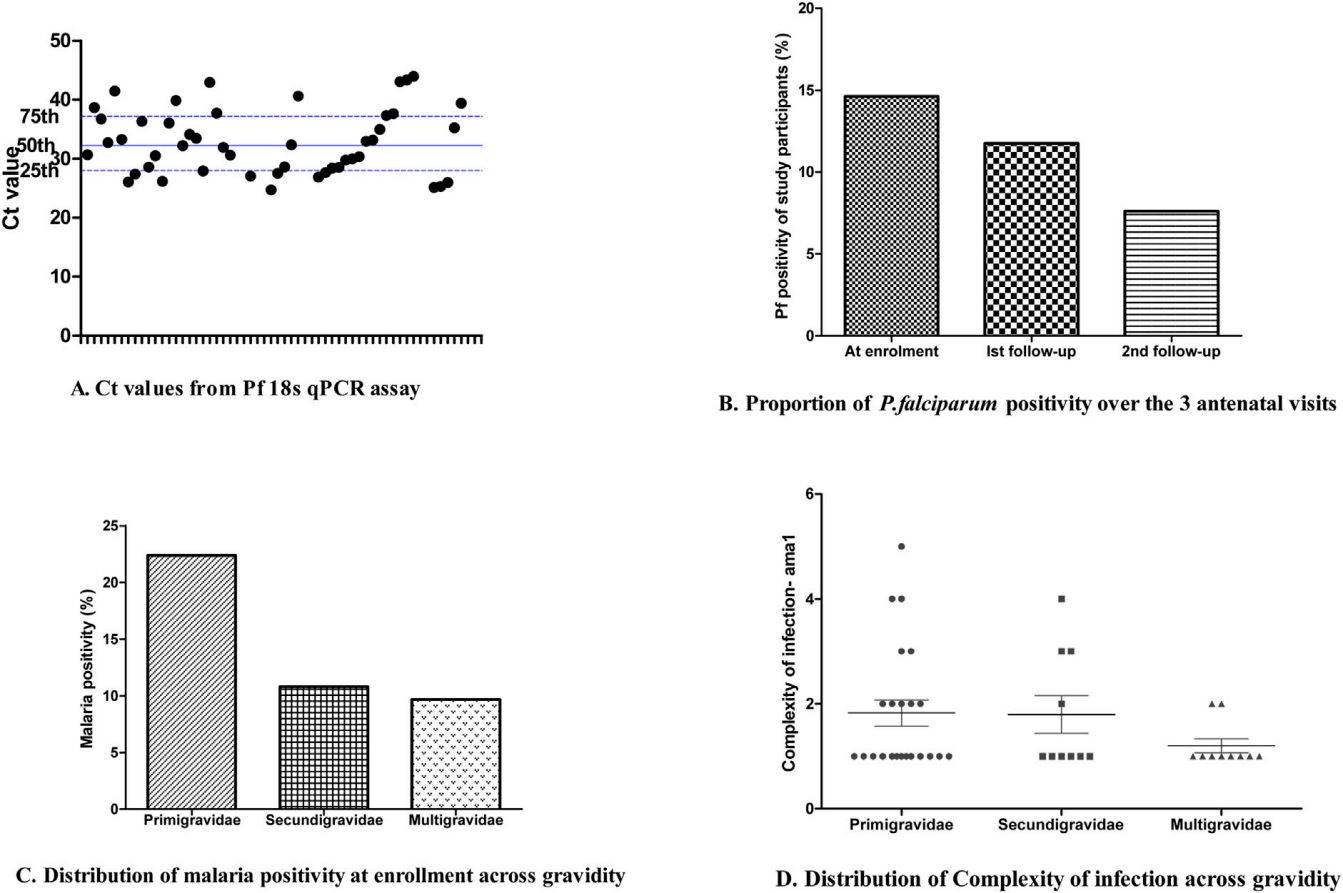
Sample *P. falciparum* positivity based on qPCR and assessed by ANC visit. **(A)** Ct values from the Pf18S qPCR assay for each sample tested. **(B)** The proportion of *P. falciparum* positivity over the three ANC visits for which samples were collected. **(C)** The distribution of *P. falciparum* positivity at enrollment based on gravidity. **(D)** The distribution of complexity of *P. falciparum* infection based on gravidity.

Out of the overall 457 samples collected for qPCR screening; 56 samples were positive following the 18S qPCR assay amplification and out of the 56 positive samples, 31 were among the samples (31/212) collected at the first contact, 18 at the first follow-up (18/153), and the remaining 7 positive samples (7/92) at the second follow-up. Five individuals whose samples were Pf18S qPCR positive at enrolment also showed malaria positivity at the first follow-up visit. In addition, three individuals whose samples were Pf18S qPCR positive at the first follow-up were also positive at the second follow-up visit. The median (IQR) Ct value was 32.2 (28.09–37.1) (Figure 1A) equivalent to a parasitemia of ∼100 parasite/μL based on the standard curve generated from the Pf3D7 laboratory isolate (Supplementary Data 1) and the proportion of positive individuals decreased over the 3 visits (Figure 1B). At enrollment, 22.4% of primigravida women (17/76) presented with *P. falciparum* infection while at the same period, 10.8% and 9.7% of secundigravida (8/74) and multigravida (6/62) women had malaria infection (Figure 1C).

Only samples with Ct <40 were selected for subsequent analyses. Of the 56 positive samples, Pfama1 amplicons were generated in 51 samples, whereas for the drug markers (*Pfdhfr, Pfdhps, Pfk13, Pfcrt* and *Pfmdr1*) 46, 41, 40, 35 and 32 amplicons were generated, respectively. All the amplicons were sequenced.

### Genetic diversity of Pfama1 within the parasite population

The ONT-targeted *ama1* sequencing approach was validated with laboratory reference isolates (Pf3D7, Pf7G8 and PfHB3) as controls. The reads matched the proportion of each of these controls were 6,072, 10,469 and 10,770, respectively. Overall, 84.3% (n = 43) of the samples were successfully sequenced for ama1 and 426,804 reads were matched to all the 43 samples, with a median (IQR) read depth of 8,275 (3,440–11,513). The mean complexity of infection (COI) based on *ama1* was 1.67 with 37% (n = 16) of the *P. falciparum* infections were polyclonal with a maximum COI of 5. Figure 1D shows the distribution of COI considering the gravidity of study participants. Seventy-two full-length *Pfama1* sequences were obtained across the 43 isolates resulting in 39 microhaplotypes, including the Pf3D7 and PfHB3 microhaplotypes (Supplementary Data 2).

### SP resistance markers

**FIGURE 2:**
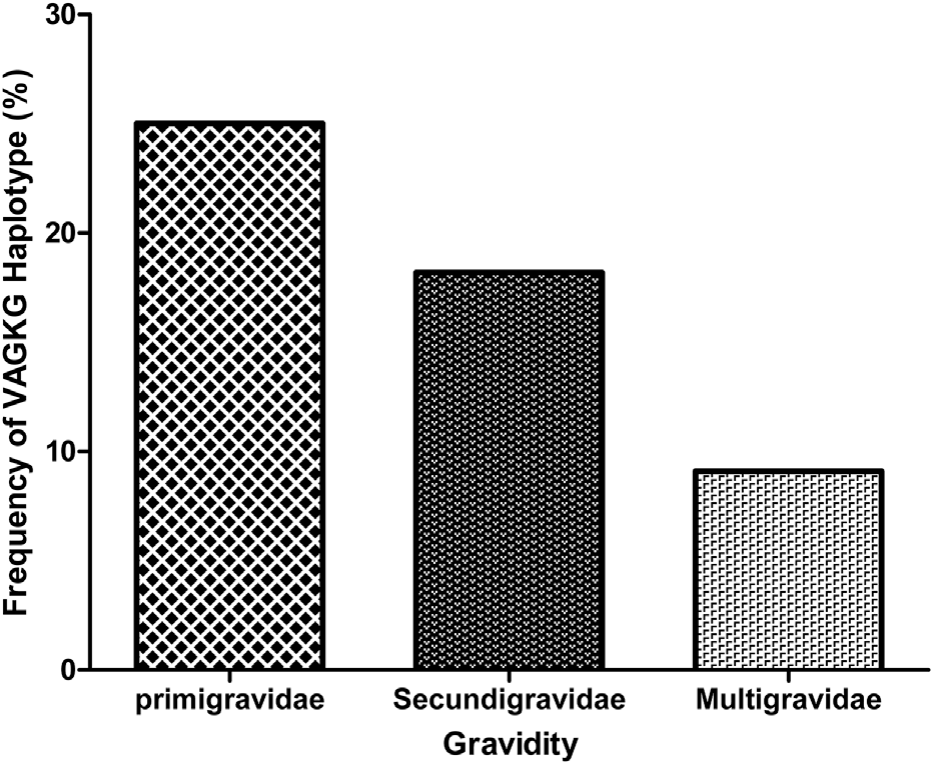
The distribution of **V**A**G**K**G** haplotype based on gravidity of study participants.

Overall, 41 (89.1%) and 37 (90.2%) samples were successfully sequenced for *dhfr* and *dhps* genes, respectively (Table 2). Within the *Pfdhfr* gene, out of the successfully sequenced samples, 90.2% (n = 37), 87.8% (n = 36) and 95.1% (n = 39) had mutations at N51I, C59R and S108N, respectively, while all were wild-type at codon 164. The **IRN**I haplotype (WHO-validated *dhfr* triple mutant for pyrimethamine resistance) was found in 81.8% (n = 36), (Table 3). For the *dhps* gene, all samples were wild-type at codon 540, while 21.6% (n = 8), 56.7% (n = 21), 89.2% (n = 33) and 27% (n = 10) had mutant alleles at codons 431V, 436S, 437G and 581G, respectively. Regarding the dhps haplotypes, 40.5% (n = 17), and 23.8% (n = 10) are ISGKA and IAGKA haplotypes, respectively, while 19% (n = 8) had the triple mutant, **V**A**GKG,** haplotype (Table 3). Out of 8 samples with **V**A**G**K**G** haplotype, five were found among the primigravida women (5/20), 2 and 1 were found among secungravida (2/11) and multigravida, respectively (Figure 2).

**TABLE 2:** The frequency of drug resistance marker mutations.

		ALL		At enrolment		At first follow-up		At second follow-up	
Gene	Mutation	Mutant	Mixed	Mutant	Mixed	Mutant	Mixed	Mutant	Mixed
		% [n]	% [n]	% [n]	% [n]	% [n]	% [n]	% [n]	% [n]
dhfr (n = 41)	N51I	90.2 [37]	2.4 [1]	100 [23]	0.0 [0]	76.9 [10]	7.7 [1]	85.7 [6]	0.0 [0]
	C59R	87.8 [36]	7.3 [3]	85.7 [18]	9.5 [2]	84.6 [11]	7.7 [1]	100 [7]	0.0 [0]
	S108N	95.1 [39]	2.4 [1]	100 [23]	0.0 [0]	84.6 [11]	7.7 [1]	100 [7]	0.0 [0]
dhps (n = 37)	I431V	18.9 [7]	2.7 [1]	0.0 [0]	5.6 [1]	23.1 [3]	0.0 [0]	66.7 [4]	0.0 [0]
	A436S	48.7 [18]	8.1 [3]	27.8 [5]	16.7 [3]	61.5 [8]	0.0 [0]	83.3 [5]	0.0 [0]
	A437G	89.2 [33]	0.0 [0]	94.4 [17]	0.0 [0]	84.6 [11]	0.0 [0]	83.3 [5]	0.0 [0]
	A581G	21.6 [8]	5.4 [2]	0.0 [0]	11.1 [2]	23.1 [3]	0.0 [0]	83.3 [5]	0.0 [0]
mdr (n = 27)	F136S	0.0 [0]	3.7 [1]	0.0 [0]	9.1 [1]	0	0	0	0
	Y184F	51.9 [14]	37.0 [10]	63.6 [7]	27.3 [3]	54.5 [6]	36.4 [4]	20.0 [1]	60.0 [3]
*crt (n = 29)	M74I	13.8 [4]	10.3 [3]	7.7 [1]	23.1 [3]	11.1 [1]	0.0 [0]	28.6 [2]	0.0 [0]
	N75E	13.8 [4]	10.3 [3]	7.7 [1]	23.1 [3]	11.1 [1]	0.0 [0]	28.6 [2]	0.0 [0]
	K76T	13.8 [4]	10.3 [3]	7.7 [1]	23.1 [3]	11.1 [1]	0.0 [0]	28.6 [2]	0.0 [0]

*For PfK13 gene, the sequence covered codons 350–590 and all were wildtype. For the Pfcrt gene, the sequence covered codons 72–76, but codons 72 and 73 were all wild type. For Pfdhfr-I164L, Pfdhps-K540E and Pfmdr-N86Y SNPs, all were wild type.

**TABLE 3:** The frequency of drug-resistant marker haplotypes in the population.

Gene	Haplotype	N	Frequency
*dhfr* (n = 44)	**IRN**I (triple)	36	81.8
	**I**C**N**I (double)	4	9.1
	N**RN**I (double)	2	4.5
	NCSI (wild-type)	2	4.5
*dhps* (n = 42)	IS**G**KA (single)	17	40.5
	IA**G**KA (single)	10	23.8
	**V**A**G**K**G** (triple)	8	19
	IAAKA (wild-type)	3	7.1
	IS**G**K**G** (double)	3	7.1
	ISAKA (wild-type)	1	2.4
*mdr* (n = 38)	N**F**	25	65.8
	NY	13	34.2
*crt* (n = 32)	CVMNK	25	78.1
	CV**IET**	7	21.9

### Polymorphisms and haplotypes in the Pfcrt and Pfmdr1 genes

For the *Pfmdr1* gene codons 86 and 184 were successfully sequenced in 27 (84.4%) samples. Three SNPs (N86Y, F136S and Y184F) were genotyped. To our knowledge, the mutation we found at codon 136 has not been previously described and it was only seen in one sample (3.7%), which requires further validation. All samples expressed the *Pfmdr1*-N86, wild-type allele. The Y184 wild type allele was observed in 3 (11.1%) samples, while 10 (37%) and 14 (51.9%) of the samples had mutant (184F) and mixed (Y184/184F) infections, respectively.

The wild-type *Pfcrt* alleles (M74, N75 and K76) were present in 22 (75.6%) samples. The mutant, 74I, 75E and 76T, alleles were found in 4 (13.8%) samples. The remaining 3 (10.3%) samples presented with mixed alleles.

## Discussion

The overall Ct value suggested a moderate level of parasitemia among the asymptomatic pregnant women. This study employed a sensitive qPCR test to detect *P. falciparum* positivity from asymptomatic participants, together with a multiplexed amplicon ONT sequencing approach to monitor the frequency of drug resistance mutations among pregnant women recruited from an antenatal clinic. The multiplexed amplicon sequencing on a MinION device offered a portable, affordable and adaptable approach for malaria molecular surveillance (MMS) similar to a recent report from another West African Country (Girgis et al., 2023).

The present findings indicated a high prevalence of asymptomatic *P. falciparum* at the enrollment (14.6%) compared to previous reports with a prevalence ranging from 4.7%–7.2% (Feleke et al., 2020; Tilahun et al., 2020; Duguma et al., 2023). It is worth noting that the previous reports were based on microscopy and RDT. A qPCR approach to detect *P. falciparum* DNA has been reported to outperform both microscopy and RDT (Opoku Afriyie et al., 2023). Our findings further revealed an increase in susceptibility to *P. falciparum* infection among women who are primigravid compared with those with repeated pregnancies (secundigravida and multigravida). This is in line with previous reports that an increase in immunity to *P. falciparum* infection that reduces level of parasitemia appears with successive pregnancies (Desai et al., 2007; Iriemenam et al., 2012).

Our findings showed that 83.9% of asymptomatic infections at enrolment were resolved through the IPTp-SP treatment. Out of the five unresolved infections at the first follow-up, none had the same ama1 COI and microhaplotype as the initial infection. However, only one ama1 microhaplotype from the second follow-up appeared the same as the initial infection. Taken together, this suggests that most of the recurrent infections were new infections rather than recrudescence. Our findings also indicate a high-level microhaplotype diversity of PfAMA1 sequences similar to a previous report in Nigerian populations (Polley and Conway, 2001).

Consistent with previous reports, our study found that the *Pfdhfr*-51**I**-59**R-**108**N** (**IRN**I), a specific microhaplotype for resistance to pyrimethamine (Naidoo and Roper, 2013), is widespread within Southwest Nigeria (Fagbemi et al., 2020; Quan et al., 2020; Adegbola et al., 2023). The more resistant **IRNL** microhaplotype was not observed. In addition, amino acid substitutions occurred in the *Pfdhps* gene at codons 431V, 437G and 581G, with no mutations at codons 436 and 540 and the frequency of **V**A**GG** infection was comparable to previous findings from Nigeria (Quan et al., 2020; Adegbola et al., 2023). Additional amino acid substitutions at codons I431V and A581G are more prominent in West Africa, but rarely occur in East Africa where the K540E is consistently predominant (Gikunju et al., 2020; Osoti et al., 2022). Exploratory analysis of our data showed that the I431V and A581G mutations were observed more frequently during the first and second follow-up visits compared to the samples collected at enrolment, suggesting selection by drug pressure.

In addition, similar to a previous report (Adegbola et al., 2023), both the I431V and A581G mutations appear to co-occur within the study population. These point mutations on the dfhr/dhps genes indicate a predominance of the sextuple mutant haplotype combination (**IRN**-**V**A**GG**). Interestingly, primigravid women have a higher frequency of VAGKG mutant haplotype indicating that 431V and 581G mutations are more likely to occur among primigravidae compared with multigravida women”. We speculate that gravidity-associated immunity might influence the carriage of 431V and 581G mutations among pregnant women with *P. falciparum* infection. Due to the small sample size in this study, further studies are suggested to investigate this hypothesis. Previous *in silico* studies suggested that these mutations could potentially affect the structure and function of the PfDHPS protein (Oguike et al., 2016). However, it remains to be determined whether emerging mutations in West Africa alter the protein function or affect the efficacy of IPTp-SP.

In our analysis, there were no mutations found in the K13 gene. This is in contrast to a recent report by Ajogbasile *et al*, that identified eight previously reported and five new SNPs on the K13 gene in children from a different population in Nigeria (Ajogbasile et al., 2022). Our findings suggest that k13 mutations are not widespread in Nigeria, and even where they occur, such SNPs have not been validated to be ACT partial resistance mediators. Similar to our findings, other African countries have also reported the absence of k13 mutations among those receiving SP-based chemoprevention (Dinzouna-Boutamba et al., 2023; Eboumbou Moukoko et al., 2023). However, recent findings from health facilities recruiting children with uncomplicated falciparum malaria, indicated that the following k13 mutations, R561H, C469Y and A675V linked to artemisinin resistance, now occur in Africa (Uwimana et al., 2020; Balikagala et al., 2021).

For the *Pfmdr1* gene, our assay only covered about 200 amino acids which featured two well-known polymorphic sites at codons N86Y and Y186F. We identified a novel SNP at codon F136S in one sample, wild-type Pfmdr1 N86Y genotype in all samples and 88.9% of the samples contained the mutant Pfmdr1 186F genotype. These findings are comparable with the reports from other parts of Nigeria and other African countries (Idowu et al., 2019; Adam et al., 2021; Hassen et al., 2022; Osoti et al., 2022). The *Pfmdr1* 86Y mutation is linked with chloroquine (CQ) and amodiaquine resistance, and its absence in Nigeria correlates with the fact that CQ drug pressure has waned following a period of withdrawal of CQ and the adoption of artemether-lumefantrine for treating uncomplicated falciparum malaria.

A low prevalence of the chloroquine-resistant CV**IET** haplotype in the present study is in contrast with previous reports. Among the samples collected among non-febrile adults, children and pregnant women in 2015, Ikegbunam *et al* (2019) reported a prevalence of 76.37% for the CV**IET** haplotype 11 years after the withdrawal of CQ. Our report suggests a decrease to 24% in the CV**IET** haplotype among asymptomatic falciparum samples collected 19 years after CQ withdrawal. This is similar to the reemergence of CQ- sensitive parasite population in East Africa where a near complete reversion to CQ-sensitive wildtype parasites has been reported (Wamae et al., 2019). The gradual reemergence of the CVMNK haplotype may signal a shift in repositioning of CQ for fighting falciparum malaria.

In conclusion, the limited molecular data of *P. falciparum* given the varying malaria endemicity across most of the country necessitates a focus on intensifying malaria molecular surveillance in Nigeria to monitor antimalarial drug resistance markers as a key component of malaria control. Chemoprevention efficacy studies (CPES) with molecular monitoring of SP resistance markers (and other drug resistance markers) as recommended by the WHO was adopted in the present study. The use of qPCR and a multiplexed amplicon ONT sequencing assays proved to be an efficient way of assessing asymptomatic *P. falciparum* infection and SP resistance genotypes in pregnant women.

## Data Availability

The datasets presented in this study can be found in online repositories. The names of the repository/repositories and accession number(s) can be found in the article/Supplementary Material.
