# Supplementary material for: ONT sequencing identifies a high prevalence of crt sensitive, triple mutant dhfr and single mutant dhps parasites within an ANC population in Nigeria
Source: Front Genet. 2024 Oct 15;15:1470156. doi: 10.3389/fgene.2024.1470156 (PMC11525066; doi:10.3389/fgene.2024.1470156)
Supplement: Supplementary file 1 [file Table1.docx]

**Supplementary data 1:** Pf18S rRNA gene qPCR standard data and curve

| **Parasite density (parasites/ul)** | **Ct Value** |
| --- | --- |
| **460000** | **18.14138** |
| **75000** | **20.80415** |
| **15000** | **24.56811** |
| **10000** | **28.30245** |
| **100** | **31.61862** |
| **10** | **35.15321** |
| **1** | **39.65703** |
| **0.1** | **43.7845** |

**B**
